# Pigment Complex, Growth and Chemical Composition Traits of Boreal *Sphagnum* Mosses (Mire System “Ilasskoe”, North-West of European Russia)

**DOI:** 10.3390/plants13172478

**Published:** 2024-09-04

**Authors:** Anastasiya Shtang, Tamara Ponomareva, Alexandra Skryabina

**Affiliations:** N. Laverov Federal Center for Integrated Arctic Research, Ural Branch of the Russian Academy of Sciences, Nikolsky Avenue, 20, Arkhangelsk 163000, Russia

**Keywords:** genus *Sphagnum*, linear increment, productivity, chemical composition, elemental composition, chlorophyll, carotenoids

## Abstract

*Sphagnum* mosses play a significant role in peat formation and carbon sequestration in mire ecosystems. It is critical to investigate the productivity and chemical composition of different *Sphagnum* species in order to assess their role in the global carbon cycle and potential in light of climate change. The data on productivity and growth characteristics during the growing season, group chemical composition and elemental composition at the beginning and end of the growing season, as well as aspects of the pigment complex operation, were collected for four *Sphagnum* species: *Sphagnum lindbergii* Schimp., *S. fuscum* (Schimp.) Klinggr., *S. divinum* Flatberg & K. Hassel, and *S. squarrosum* Crome. High cover density and productivity, low ability to decompose, and constancy of the pigment complex of *S. fuscum* reflect a high degree of adaptation to the specific conditions of ridges. A constant chemical composition of *S. lindbergii* during the growing season can be explained by stable conditions of hollows that allow it to maintain its metabolic processes, but the light conditions in hollows bring the reaction of the pigment apparatus of this species closer to shaded *S. divinum* and *S. squarrosum*. *S. lindbergii* and *S. squarrosum* contain more nitrogen than other species and have a greater ability to decompose.

## 1. Introduction

The mosses of *Sphagnum* L. are a special group of bryophytes distinguished from other mosses by the special differentiation of shoots in terms of anatomy and cell morphology [1]. *Sphagnum* mosses are ecosystem drivers in mires, accounting for 25–30% of the boreal biome [2,3]. *Sphagnum* mosses form peat on oligotrophic mires, under the influence of low pH and anoxic oligotrophic conditions, resulting in a large amount of atmospheric carbon being fixed in the organic matter of mosses, and deposited into a peat deposit [4]. However, despite the importance of mosses in global biosphere processes, moss ecology and physiology have received less attention than that of other plant groups [5]. The difficulties of identifying mosses in the field, as well as their small size, make it challenging to create adequate sampling or repetition in tests. Even when mosses form large colonies, they are frequently intermingled with other moss species, tracheophytes, animals, and fungi [6,7].

The genus *Sphagnum* includes several hundred species spread over the globe in a range of habitats, but the primary need for the survival of *Sphagnum* mosses is the availability of accessible water [8,9]. *Sphagnum* mosses, as poikilohydric plants, cannot withstand prolonged drying and regulate the water exchange of their tissues [10]. The natural distribution of *Sphagnum* mosses according to their ecological optimums occurs along the topographic gradient, depending on the physical-chemical properties of the water, the level of the water table, and the humidity of the substrate surface [11,12]. While tracheophytes were able to develop anatomical structures and specialized tissues to respond to environmental changes, mosses adapted to deal with physiological stress only by regulating metabolism [13]. Thus, *Sphagnum* mosses metabolism, photosynthetic activity, and the intensity of growth processes are all affected by fluctuations in environmental conditions, particularly when the seasons change [14].

The productivity of different *Sphagnum* species varies widely, ranging from 260 to 1450 g m^−2^ yr^−1^ [15]. The production of organic matter by *Sphagnum* mosses as well as its chemical composition are both drivers of peat formation and the result of carbon fixation from the atmosphere [16,17]. So, understanding the seasonal changes occurring in the sphagnum cover as well as inter-species differences will allow the assessment of moss participation in the global carbon cycle and the production potential of selected species of *Sphagnum* in the context of climate change. Information on ecophysiological features of *Sphagnum* mosses is important because of the ecological plasticity of some species and the proven presence of interspecies competition under the changing properties of the edafotope, which impacts the possible composition of plant communities in a changing climate [18,19]. Furthermore, the specifics of the practical use of *Sphagnum* mosses as raw materials (for example, projects including such land use as sphagnum farming) are determined by their composition and properties, which must be explored [20,21].

Since *Sphagnum* mosses grow in different conditions of water availability, light, and heat supply, it is reasonable to form a hypothesis: growth and production capabilities, and features of chemical and elemental composition, as well as the nature of their features of seasonal dynamics, vary between *Sphagnum* species. Thus, the aim of the study was to investigate the growth, chemical composition, and pigment complex features of four species (*Sphagnum lindbergii* Schimp., *S. fuscum* (Schimp.) Klinggr., *S. divinum* Flatberg & K. Hassel, and *S. squarrosum* Crome) in different habitats of the boreal ombrotrophic mire system, including seasonal variations.

## 2. Results

### 2.1. Growth and Production Characteristics

**Figure 1:**
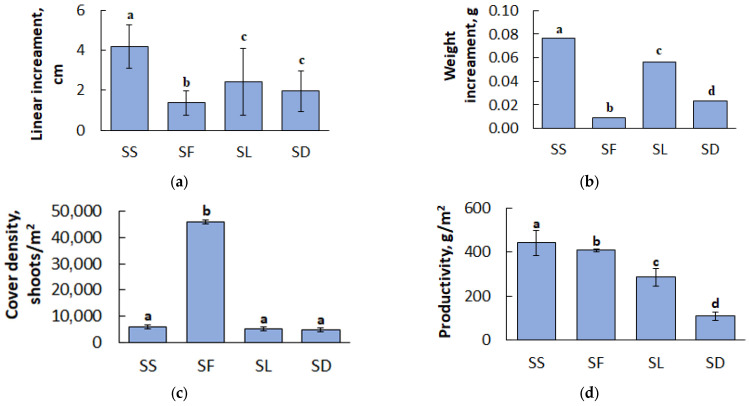
The linear increment (**a**), weight increment (**b**), density of *Sphagnum* shoots (**c**) and productivity (**d**) of *Sphagnum* species during the vegetation season of 2023. Identical letters denote no statistically significant difference (*p* > 0.05).

The results of the determination of linear and weight increments of *Sphagnum* mosses, obtained from May to October 2023, are shown in Figure 1a,b. *S. squarrosum* had the highest mean linear and weight increments, reaching 4.18 cm and 0.0764 g, respectively. The lowest linear and weight increments were found for *S. fuscum*: 1.38 cm and 0.0089 g, respectively. The linear increment for the species *S. divinum* and *S. lindbergii* didn’t significantly differ (1.96–2.43 cm), and the mean weight increment of these species was 0.0235 g and 0.0563 g, respectively.

The results of the *Sphagnum* cover density determination for the studied species are shown in Figure 1c. The data show that there are no statistically significant differences in the number of shoots per square meter of habitat among large loose growing species of *Sphagnum* mosses (*S. lindbergii*, *S. divinum*, and *S. squarrosum*) with cover density ranging from 4667 to 5767 shoots/m^2^. *S. fuscum* had the highest cover density among all species (46,000 shoots/m^2^).

The results of the *Sphagnum* mosses productivity calculation are shown in Figure 1d. The productivity of the *Sphagnum* species varies. In 2023, the mean productivity for the studied species was as follows: *S. squarrosum*—441.2 g/m^2^; *S. fuscum*—407.0 g/m^2^; *S. lindbergii*—285.1 g/m^2^; and *S. divinum*—109.4 g/m^2^.

### 2.2. Elemental Composition

The CHN analysis of the studied *Sphagnum* mosses revealed modest interspecies heterogeneity in elemental content (Table 1). For all species, statistically significant differences were found between carbon content in summer and autumn, but no such differences were found for hydrogen content. *S. lindbergii* and *S. squarrosum* had statistically significant differences between summer and autumn nitrogen content. The C/N ratio showed greater interspecies variability, ranging from 34 to 63.

### 2.3. Chemical Group Composition

Table 2 shows the share of different groups of organic matter in *Sphagnum* mosses. The group chemical composition of studied *Sphagnum* mosses varied by species and over the growing season (Table 3). For all species, statistically significant differences were found between the water-soluble fraction in June and September. The hemicellulose fraction and lipid fraction percentage of *S. fuscum* and *S. divinum* decreased from June to September, but for *S. lindbergii,* statistically significant differences weren’t found between June and September values. Species and seasons had no effect on the fraction of pectin compounds. The proportion of lignin-like compounds, regardless of species, decreased from June to September. The cellulose content increased from summer to autumn in all species. By autumn, ash content in samples of *S. fuscum* and *S. divinum* had declined. 

### 2.4. Photosynthetic Pigments

Table 4 shows the dynamics of the pigment complex for four studied species of *Sphagnum* mosses from May to October. The data dispersion for all tested parameters varied according to *Sphagnum* species and sample month (Table 5). The distribution of chlorophyll *a* (Chl *a*) concentrations during the study did not differ between *S. lindbergii* and *S. squarrosum*. Chlorophyll *b* (Chl *b*) and carotenoids (Car) concentrations did not differ between three species, *S. lindbergii*, *S. divinum*, and *S. squarrosum*. The concentration of photosynthetic pigments in *S. fuscum* did not differ by month during the study period. For *S. lindbergii* and *S. divinum*, the content of all photosynthetic pigments reached maximum values in August. *S. squarrosum* had the highest chlorophyll concentration also in August and the lowest in another time period—in June. Carotenoids in *S. squarrosum* remained minimal in June and July but peaked in August.

The Chl *a*/*b* ratio values for *S. lindbergii* and *S. divinum* were distributed similarly. *S. squarrosum* had a higher Chl *a*/*b* ratio, while *S. fuscum* had the lowest ratio. Only *S. squarrosum* showed a noticeable maximum and minimum in the Chl *a*/*b* ratio (maximum in September, minimum in June).

The Chl/Car ratios for both *S. squarrosum* and *S. lindbergii* did not differ by month. *S. fuscum* had the highest Chl/Car ratio values in October and the lowest in June. For *S. divinum*, the maximum Chl/Car ratio was observed in August and the lowest in June. The distribution of chlorophyll share in the light-harvesting complex (LHC) did not differ considerably between *S. lindbergii* and *S. divinum*. The chlorophyll share in LHC for *S. fuscum* had the narrowest range. *S. squarrosum* had a lower chlorophyll share in LHC than other species. The latter species experienced a significant peak in July and a minimum in September.

In addition to the pigment complex data, Table 4 shows the relative water content (RWC) of moss samples for studied species, which was evaluated concurrently with pigment extraction. The relative water content of *Sphagnum* mosses varied during the growing season: RWC in *S. lindbergii* varied in the range of 1820–2606% (minimum in July), in *S. fuscum*—855–1030% (minimum in August), in *S. divinum*—546–1328% (minimum in July), and in *S. squarrosum*—733–2190% (minimum in August).

### 2.5. Dependence of Pigment Complex on Environmental Factors

During the study, the correlations between the parameters of the *Sphagnum* moss pigment complex (Chl *a*, Chl *b*, Car, Chl *a*/*b*, Chl/Car, LHC) and the environmental conditions (air temperature, sum of precipitation, bog water level (BWL), photoperiod, RWC) were investigated (Table 6). It has been proven that environmental conditions influence the pigment complexes of studied species of *Sphagnum* mosses. The chlorophyll and carotenoids content of *S. lindbergii* correlates positively with the mean air temperature for 10 days preceding the day of moss sampling and negatively with the bog water level. The Chl/Car ratio in this species correlates with the relative water content of moss.

In *S. divinum*, pigment content correlates positively with air temperature and negatively with bog water level, similar to *S. lindbergii*. Furthermore, the Chl/Car ratio correlates with air temperature and bog water level. Precipitation also has a negative correlation with chlorophyll concentration and the Chl/Car ratio. The Chl *a*/*b* ratio and the share of chlorophyll in the light-harvesting complex in *S. divinum* are related to day length and relative water content in moss tissues.

The concentration of chlorophylls in *S. fuscum* is related to air temperature and bog water level. Carotenoids content corresponds with precipitation amount, and the Chl/Car ratio correlates with day length.

The pigment concentration in *S. squarrosum* has a negative correlation with daylight length and moss relative water content. The Chl *a*/*b* ratio and the fraction of chlorophyll in the light-harvesting complex are related to air temperature and daylight length.

## 3. Discussion

Nowadays, a large amount of information about the growth and productivity of *Sphagnum* mosses has been gathered, and the main growth patterns of *Sphagnum* mosses from different ecological groups have been determined [22,23,24]. However, assessing the production and growth properties of *Sphagnum* mosses can be the first step in ecophysiological study [25]. It serves as the foundation for selecting subjects for the research of moss ecophysiology, as plant primary products are the consequence of metabolic processes in plant organisms [26]. Differences in biomass growth and accumulation rates are the main and most obvious markers of differences between *Sphagnum* mosses [25,27]. Moss growth patterns are determined by their adaptation to successful photosynthesis in the circumstances in which they live [28]. *S. fuscum*’s dense turf and tiny shoot size contribute to a reduction of the area of the moss’s light-absorbing surface, preventing photo-damage. In contrast, growing in forested and watered conditions, large *Sphagnum* species do not block sunlight from entering the turf [29].

The results obtained for linear and weight increment, sphagnum cover density, and productivity are consistent with studies conducted in boreal mires by other researchers, although some indicators, such as moss cover density, can vary in one species over a wide range [23]. As expected, the results indicated that mesotrophic *S. squarrosum* has the highest productivity between species under investigation (441.2 g/m^2^). However, *S. fuscum*, the slowest and the most densely growing species, was the second most productive (407.0 g/m^2^) due to its projective coverage. The weight increments varied across all *Sphagnum* species chosen as study targets, revealing the most obvious species variations. It is likely that for a quick assessment of the production potential of sphagnum mosses, further research may be limited to the least time-consuming method of measuring linear increments, with further determination of their weight.

The group chemical composition of the organic matter of *Sphagnum* mosses is identical to that of tracheophytes; the only difference is the group ratio. Existing research shows that the chemical composition of mosses and its variations are influenced by both the species’ taxonomic affiliation and environmental factors [30]. Cellulose, hemicellulose, and pectin are the primary components of moss cell walls, accounting for up to 85% of dry mass [31]. *S. fuscum* and *S. lindbergii* that grow on oligotrophic mires had a slightly lower hemicellulose and ash content and greater cellulose content than *S. divinum*, which grows in a pine forest on the edge of the mire. In the tissues of the studied species, the content of water-soluble compounds was several times lower in September than in June. A high content of water-soluble compounds in mosses in June may imply strong metabolic activity during this time. The decrease in content of water-soluble compounds by autumn implies that these compounds, together with lipids, are consumed for growth activities and recovery after drying, which was observed in all of the mosses tested throughout the study [14]. It is worth mentioning that in the aquatic species *S. lindbergii*, the lipid content remained constant throughout the growing season. In general, seasonal changes in the share of groups of compounds in the composition of organic matter for *S. fuscum* and *S. divinum* occurred concurrently, whereas the share of some groups in *S. lindbergii* was constantly lagging behind, probably due to the fact that the conditions of the hollows are considered to be quite stable [14].

When analyzing the chemical composition of mosses in terms of their potential to degrade, it is important to note that the proportion of pectins, hemicellulose, cellulose, and lignin-like compounds represents a plant species’ anticipated rate of decomposition [21,32,33]. *Sphagnum* tissues high in lignin-like compounds and low in nitrogen are considered biochemically resistant to breakdown [34]. A 12–15% decrease in the amount of lignin-like compounds by autumn in all studied species could imply a decline in degradation resistance [35]. This is especially obvious for the hypohydrophyte species *S. lindbergii* and *S. squarrosum*, which have higher nitrogen content. Another measure that correlates with plant resistance to degradation is the C/N ratio, which increases from eutrophic to oligotrophic species [36]. The highest values of this indicator show *S. fuscum* and *S. lindbergii*, that grow in ombrotrophic circumstances. *S. divinum* and *S. squarrosum* are more mesotrophic, with lower C/N ratios.

Changes in moss chemical composition have also been linked to growth processes. Seasonal fluctuations in the concentration of organic matter fractions in moss tissues are caused by species-specific genetic features and environmental changes. Seasonal variations in growth rate are intricately connected to fluctuations in the intensity of main and secondary metabolic processes [37]. At the same time, photosynthetic intensity is not always proportional to growth intensity [38]. The most intensive growth processes in mosses are likely to occur in spring and autumn, when the air temperature is not too high and moisture levels are enough [37]. In wetter environments, *Sphagnum* mosses’ active growth periods may be longer. For example, the increase in nitrogen content in *S. lindbergii* and *S. squarrosum* could be attributed to the translocation of nitrogen from old tissues into young tissues during the active growth period in autumn, because mosses growing in humid environments have more opportunities and time for growth processes [39].

The ability of the pigment system to modify and adapt is critical for the stability of the plant organism [40]. The pigment apparatus has been adapted to include the content of antenna pigments (Chl *b*, Car), photoprotective pigments, and chlorophyll in LHC. The concentration and ratio of photosynthetic pigments are used to assess plant physiological condition and photosynthetic activity [38]. Among the studied species of *Sphagnum*, more pigments were accumulated by species that were not exposed to excessive insolation: *S. squarrosum* and *S. divinum*, and by *S. lindbergii* growing in water. These three species have larger ratios of Chl *a*/*b* and Chl/Car than *S. fuscum*, which grows on open mire ridges and accumulates little pigment but has a high relative level of photoprotective carotenoids. The presence of more than 50% chlorophyll in LHC is an adaptation to low light [41], but high amounts have been observed in all investigated species, including *S. fuscum*. It is known that the photosynthesis rate in *Sphagnum* mosses decreases after drying periods because it is dependent on the mosses’ water content, and the maximum photosynthesis intensity is usually achieved at the end of the growing season, when the air temperature and precipitation are optimal for photosynthesis of plants with the C3-pathway [40]. Although the relationship between the photosynthetic pigment concentration and the photosynthesis intensity is ambiguous and inconsistent, some evidence suggests that the Chl *a* content may reflect the photosynthetic potential of the plant [42]. Based on this assumption, we can conclude that the mosses *S. lindbergii*, *S. divinum*, and *S. squarrosum* are most photosynthetically active in August. The pigment content of *S. fuscum* has not changed, so it is difficult to predict the evolution of the photosynthetic activity of this species by proxy. Only in *S. squarrosum*, chlorophyll *a* was correlated negatively with RWC. For the other two species, the maximum chlorophyll *a* concentration was observed after the period with lowest values of RWC.

According to the results, high temperatures reduce pigment content, while rising water table levels and precipitation lead to enhanced pigment concentration in *S. fuscum*, which grows in water-stressed environments with high insolation. The probability of photodamage increases within the long daylight characteristic of high latitudes [42], hence the length of the photoperiod correlates directly with Chl/Car. Although water is required for moss activity, excessive water reduces photosynthesis [14]. For hollow-growing *S. lindbergii*, this constraint manifests itself as a negative correlation of pigment content with the water table level and a positive correlation of Chl/Car ratio to RWC. Interestingly, when *S. divinum* grows away from the water table, the link between pigment concentration, water table level, and precipitation is comparable to that reported in *S. lindbergii*. The association between air temperature, precipitation, water table level, and the Chl/Car ratio in *S. divinum* suggests that this species does not require a high water content to maintain an effective level of photosynthesis. Simultaneously, RWC influences moss response to light conditions: high RWC enhances antenna pigment synthesis and redistribution among photosystems [38]. Daylight length has a similar influence on the pigment complexes of this species and *S. squarrosum*. Most likely, in forested areas, mosses are not subject to the negative effects of extended daylight hours and continue to actively capture light with the help of antenna pigments.

## 4. Material and Methods

### 4.1. Study Area

The study was conducted in 2023 on a wetland area 55 km south of the Dvina Bay of the White Sea, in Arkhangelsk, in the European part of Russia (64°19′47.02″ N; 40°37′10.22″ E). The climate of the research area is temperate-continental subarctic, with significant influence of the Arctic Ocean seas. The weather station “Arkhangelsk” of Russia’s Northern Directorate of Hydrometeorological Service (64°31′37.51″ N; 40°40′41.09″ E) provides data on the study area’s climatic conditions. The weather station is located 22.5 km from the study area, at an elevation of h = 8 m above sea level. The mean annual temperature in the study region is 1.9 °C. The mean temperature of the coldest month (January) is −11.6 °C; of the warmest month (July) is 16.5 °C. The mean annual precipitation in the study area is 634 mm; during the warm season (May–October) nearly half of the annual precipitation falls. The number of days with precipitation per year is 263 days. The number of days with stable snow cover per year is 180 days. The mean annual maximum depth of snow cover is 102 cm. The warm period (with a mean day temperature above 5 °C) lasts for 120–150 days. The maximum length of daylight is 21 h 32 min (20 June), and the minimum is 3 h 54 min (20 December).

The study sites were established within the boundaries of the extensive ombrotrophic mire system “Ilas”. Such mire systems, along with boreal coniferous forests, are the environmental core of the boreal biome. The mire system mostly consists of *Sphagnum*-dominated domed mires with concentric hummock-hollow patterning and an ombrotrophic type of nutrition. Pine-*Sphagnum* forests and meso-eutrophic sedge-*Sphagnum* mires with deciduous trees developed at the margins of the mire system. Study sites 1 and 2 were established in the central part of the *Sphagnum*-dominated domed mire on a hummock in the pine-shrub-*Sphagnum* community (1), as well as in the adjacent *Scheuchzeria*-*Sphagnum* hollow (2). Study site 3 was established at the ombrotrophic bog margin in the pine-*Sphagnum* forest. Study site 4 was also established at the ombrotrophic bog margin at the meso-eutrophic sedge-*Sphagnum* mire.

### 4.2. Weather and Climate Data

Data on the 2023 air temperature, precipitation, and daylight duration in the study area necessary for data discussion and statistical analysis were obtained from http://meteo.ru/ (accessed on 23 April 2024) and https://world-weather.ru/ (accessed on 23 April 2024) (from the “Arkhangelsk” weather station). In addition, water table level was measured during the warm season (May–October) in the hydrological wells at all study sites (Table 7).

### 4.3. Focal Species

Four species of the genus *Sphagnum* L., common in the boreal mire complexes, and belonging to different sections of the genus, have been selected as targets, suggesting anatomomorphological differences between them (Table 7). There are *Sphagnum lindbergii* Schimp. (section Cuspidata), *Sphagnum fuscum* (Schimp.) Klinggr. (section Acutifolia), *Sphagnum divinum* Flatberg & K. Hassel (section Sphagnum), and *Sphagnum squarrosum* Crome (section Squarrosa) (Table 8). The selected species are found in different habitats, and therefore exist under different luminosities and hydrothermal conditions.

**Figure 2:**
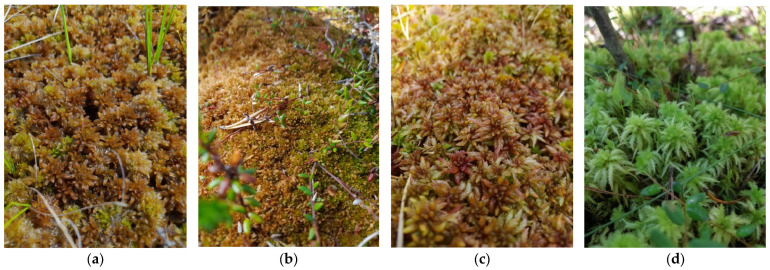
Focal species: (**a**) *Sphagnum lindbergii* Schimp.; (**b**) *Sphagnum fuscum* (Schimp.) Klinggr; (**c**) *Sphagnum divinum* Flatberg & K. Hassel; (**d**) *Sphagnum squarrosum* Crome.

*Sphagnum lindbergii* is a species involved in the formation of vegetation in various types of hollows in ombrotrophic or mesotrophic mire. The shoots are large and brown in color. This species is well distinguished in ombrotrophic hollows by the dark color of the stems [46]. This species is characteristic of the northern boreal mire flora and is not found further south (Figure 2a) [47]. *Sphagnum fuscum* is the main ecosystem driver and peat-forming plant of ombrotrophic mires, dominating on ridges and hummocks, where it grows together with shrubs and lichens [46]. The shoots are small, brown or greenish brown, forming dense cover that is difficult to separate into individual shoots (Figure 2b) [46]. *Sphagnum divinum* occurs everywhere in the northern boreal mires and grows both on hummocks with abundance of shrubs and on the margins of ombrotrophic mires. The shoots are large, with noticeable imbricated leaves, purple-red to brown-red (Figure 2c) [48]. *Sphagnum squarrosum* is a widespread forest species. It grows, most often, in wet spruce forests, in habitats where willow, birch, or alder are growing in vegetation cover; it often coexists with sedges and *Menyanthes trifoliata* [47]. It has big, green shoots with spiky-looking branches (Figure 2d) [46].

### 4.4. Growth and Production Characteristics

The most optimal methods for studying linear growth were selected depending on the characteristics of the turf formed by different types of *Sphagnum* mosses [49]. *S. fuscum* forms dense turfs that provide capillary rise of water from the depth of the peat deposit and reduce evaporation [28]. Violation of the integrity of such turf can lead to drying out of individual shoots and termination of growth processes due to the low desiccation tolerance of *S. fuscum* [50]. Thus, methods of bandaging or cutting to a known length are not suitable for measuring the linear growth of *S. fuscum*. So, we chose the least invasive outer marks method: for *S. fuscum*, the linear increment was determined by placing wooden sticks with moss capitula marks in the turf in May in order to mark the level of the moss heads again in the autumn [25]. The distance between the marks made for *S. fuscum* was measured for 208 wooden sticks.

Wooden sticks must be positioned motionless in the moss cover, thus it is not suitable for measurement of the linear increment of species that do not form dense turf (*S. lindbergii, S. divinum,* and *S. squarrosum*). For studying these species, the method of cutting up to a known length was used [25]. In May, moss shoots selected from natural habitats were cut to 5 cm in length and then formed into loose bundles of 10–15 plants. The formed bundles were placed back in the moss cover, from where the shoots were removed. At the end of September, each shoot was measured, and the difference between the initial and final length determined the linear increment during the growing season. For *S. lindbergii,* 60 shoots were measured, for *S. divinum,* the number of measured shoots was 58; and for *S. squarrosum,* there were 110 shoots. 

The units of length of different parts of the stem (capitula, upper stem, lower stem) have different masses and researchers use different approaches to calculation based on linear and mass increment measurements [24,51,52]. Therefore, for the calculation of mass growth for this study, we chose a conventional unit—a section of the shoot is 1 cm long, including the head and part of the stem. To calculate the weight increment, the dry mass of 1 cm-cut (*n* = 50) of the shoots from the moss capitulas of each studied species was multiplied by the linear increment. In order to estimate the density of the *Sphagnum* cover, the number of moss capitulas in a space bounded by a 10 cm × 10 cm frame was calculated fivefold on each study site. To calculate the productivity of each studied species of moss during the growing season, the weight increment and *Sphagnum* cover density values were used [24].

### 4.5. Chemical Composition

Parts of the moss shoots, visually identified as alive, were taken to determine the fractional composition of the organic matter, ash content and the CHN content at the beginning and end of the warm period. The group chemical composition of organic matter was analyzed for *S. lindbergii*, *S. fuscum,* and *S. divinum*. CHN analysis was performed for all species. At the study sites, a mixed moss sample was taken (about 300 g) that contained samples of a certain species of moss collected in different locations of one population. The sampling was carried out by using the “envelope” sampling method (*n* = 5). In the laboratory, the mosses were dried and sieved through a sieve with a 2.0 mm mesh. Three samples for chemical analyses were taken randomly from a dried mixed sample. Six fractions of organic matter were extracted sequentially from dried and crushed *Sphagnum* mosses by various solvents. After extraction of the water-soluble fraction, pectin substances, hemicellulose fraction, and cellulose fraction, the solvent was removed by rinsing from the *Sphagnum* residue on a glass or paper (for water-soluble fraction). The filters were preliminarily dried to a constant mass in a drying oven at 105 °C. The share of groups of substances was calculated based on the loss in mass of samples after extraction [53].

#### 4.5.1. Lipid Fraction 

*A Sphagnum* sample in a paper cartridge (*n* = 2 for every species) was placed in the extractor of the Soxlet apparatus. Acetone was poured into the extractor of the apparatus in a volume equal to 2 volumes of the extractor of the Soxlet apparatus. The Soxlet apparatus was heated in a water bath at 50–60 °C. Extraction continued until the solvent was completely discolored. Portions of extract (15 mL) were placed in flasks (*n* = 2 for every paper cartridge) with a known dry mass. Flasks with an extract were left in air for 24 h to remove solvent residues and then dried to a constant mass in a drying oven at 105 °C. The mass of lipid fraction was converted to absolutely dry organic matter [53].

#### 4.5.2. Water-Soluble Fraction 

The water-soluble fraction (simple sugars and phenolics) was extracted from lipid-free residue. Extraction was performed three times using distilled water in a ratio of 1:30, 1:21 and 1:15 by heating on a water bath at 80 °C for 2, 1.5 and 0.5 h, respectively (*n* = 3) [53].

#### 4.5.3. Pectin Substances

After the separation of water-soluble substances from *Sphagnum* samples, a fraction of pectin substances was extracted three times by a mixture of 0.5% oxalic acid and ammonium oxalate solutions in a ratio of 1:30, 1:21, and 1:15 on a boiling water bath for 2 h (*n* = 3) [54].

#### 4.5.4. Hemicellulose Fraction

The fraction of hemicellulose was extracted from moss samples twice by a 10% NaOH solution in a ratio of 1:30 for 1 h (*n* = 3) [54].

#### 4.5.5. Cellulose and Lignin-like Compounds

The cellulose was extracted by an 80% H_2_SO_4_ solution from the samples of studied *Sphagnum* mosses in a ratio of 1:30 for 1 h; thereafter, the acid solution was diluted with distilled water to a five percent concentration and the extraction continued on a boiling water bath for 1 h (*n* = 3). The non-hydrolyzed residue after extraction was assumed to be lignin-like compounds [53].

#### 4.5.6. CHN and Ash Content

Total carbon, hydrogen, and nitrogen content was determined using the EuroVector EA-3000 element analyzer (Eurovector, S.p.A., Pavia, Italy) (*n* = 3). Based on the data obtained, the C/N ratio was calculated. The ash content of moss samples was determined by using the LOI method in a muffle furnace LF-9/11-V1 (LOIP, Moscow, Russia) at t = 800 °C (*n* = 3).

### 4.6. Phothosynthetic Pigments and Relative Water Content

Chlorophyll *a* (Chl *a*), chlorophyll *b* (Chl *b*), and carotenoids (Car) were determined once a month from May to October in the upper photosynthetically active parts of shoots, which, according to the literature, are 1.8 cm long in *S. fuscum* and in other, loose-growing species are 3 cm long [28]. At the study sites, a mixed moss sample (about 100 g) was formed in the same way as for chemical analysis, but the moss was not dried. Extraction was done with 80% acetone solution (*n* = 3) for every species in one analysis. An extract containing a mixture of chlorophylls and carotenoids was analyzed using a spectrophotometer UV-1800 (Shimadzu, Kyoto, Japan) at wavelengths 470, 663.2, and 646.8 nm. The pigment concentrations were calculated taking into account the absorption coefficients proposed in Lichtenthaler, 1987 [55]. The pigment content was recalculated into a dry mass of plant material. In addition to pigment concentrations, their ratios (Chl *a*/*b* and Chl/Car) and the proportion of chlorophylls in the light-harvesting complex (LHC) were calculated [56]:

LHC = (Chl *b* + 1.2 Chl *b*)/(Chl *a* + Chl *b*) (1)

The relative water content (RWC) in moss tissues was determined once a month (when the chlorophyll concentrations were determined) by drying at 105 °C. RWC was calculated based on percentage of water relative to dry mass [57].

### 4.7. Statistical Analysis

The obtained data on production, growth, and chemical indicators of *Sphagnum* mosses are given in the article in the form of the mean value ± standard deviation value. The parameters of the pigment complex and the group chemical composition of the organic substance of the studied *Sphagnum* mosses were analyzed using generalized linear mixed models (GLMM) with post-hoc Tukey test [58]. In the first case, the species and month were chosen as fixed factors; in the second, the species and season. To compare the samples obtained, the Kruskal-Wallis criterion was applied with the U Mann-Whitney posterior criterion. The Spearman’s correlation coefficient was used to identify the relationship between the pigment complex parameters and environmental conditions. The parameters included in the analysis were the following: mean temperature for 10 days before sampling, sum of precipitation for 10 days before sampling [59], water table level, length of daylight, and relative water content in mosses. Data processing was performed in SPSS 11 [60].

## 5. Conclusions

The obtained correlation values of the parameters of the pigment complex of *Sphagnum* mosses and the characteristics of the microclimate indicate a special interaction of each *Sphagnum* species with habitat conditions. However, our findings revealed that the result of this interaction—the characteristics of growth processes, chemical composition, and pigment complexes—are not absolutely species-specific, but they help to classify *Sphagnum* mosses into groups that differ from those formed on the basis of the trophic or moisture factor. *S. fuscum,* the most common species of ombrotrophic bogs, demonstrated unique features (cover density and pigment complex) reflecting the high degree of adaptation to specific conditions of ridges. High productivity, dominance in the structure of communities, and low ability to decompose make *S. fuscum* an important element of the carbon cycle of bogs. Stable drainage conditions of hollows allow *S. lindbergii* to maintain a constant chemical composition during the growing season, but the light conditions in the aquatic environment bring the reaction of the pigment apparatus of this species closer to *S. divinum* and *S. squarrosum* growing under forest canopy. In addition, *S. lindbergii* and *S. squarrosum*, despite the oligotrophy of *S. lindbergii*, contain more nitrogen than *S. fuscum* and *S. divinum* and have a greater ability to decompose. Further research of species included in the same sections and generally acknowledged ecological categories as previously investigated will provide additional information about the functioning and biosphere role of complex *Sphagnum* communities and *Sphagnum* moss-dominated communities.

## Figures and Tables

**Table 1:** Content of carbon, hydrogen, nitrogen (%), and C/N ratio in the studied moss species at the beginning and end of the growing season in 2023 (*n* = 3).

Species	Period of Sampling	C	H	N	C/N
*S. lindbergii*	June	37.62 ± 0.009	6.40 ± 0.036	0.70 ± 0.021	54
	September	45.69 ± 0.0073 *	6.03 ± 0.003	0.90 ± 0.002 *	50
*S. fuscum*	June	38.61 ± 0.027	6.24 ± 0.095	0.77 ± 0.003	50
	September	45.43 ± 0.032 *	5.88 ± 0.064	0.72 ± 0.007	63
*S. divinum*	June	37.79 ± 0.007	6.01 ± 0.098	0.98 ± 0.011	39
	September	45.65 ± 0.007 *	6.02 ± 0.002	1.01 ± 0.014	45
*S. squarrosum*	June	36.40 ± 0.046	6.11 ± 0.15	0.92 ± 0.012	39
	September	45.30 ± 0.053 *	6.13 ± 0.006	1.31 ± 0.009 *	34

* Asterisks denote statistically significant differences between June and September values (U Mann-Whitney test, *p* < 0.05).

**Table 2:** The organic compounds content (%) in *Sphagnum* species at the beginning and end of the growing season 2023 (*n* = 3).

Fraction of Organic Compounds	Month	*S. lindbergii*	*S. fuscum*	*S. divinum*
Water-soluble fraction	June	27.01 ± 1.11 a	17.72 ± 6.56 a	26.76 ± 0.79 a
	September	10.26 ± 2.90 *b	5.88 ± 0.19 *b	9.56 ± 0.25 *b
Hemicellulose fraction	June	23.25 ± 1.10 b	29.75 ± 1.89 a	30.69 ± 0.26 a
	September	22.33 ± 1.14 d	21.52 ± 0.94 *cd	17.45 ± 2.44 *c
Pectin substances	June	3.72 ± 0.01 a	2.75 ±0.14 a	2.58 ± 1.18 a
	September	2.96 ± 0.78 b	1.23 ± 0.10 b	2.32 ± 2.12 b
Lipid fraction	June	1.93 ± 0.02 a	2.25 ± 0.21 a	3.58 ± 0.15 b
	September	2.16 ± 0.29 cd	1.52 ± 0.16 *d	2.78 ± 0.10 *c
Lignin-like substances	June	14.05 ± 0.53 a	17.52 ± 0.76 a	15.32 ± 1.58 a
	September	1.73 ± 0.16 *b	1.80 ± 0.02 *b	2.00 ± 0.18 *b
Cellulose	June	30.09 ± 0.75 b	30.02 ± 0.49 ab	21.26 ± 2.18 a
	September	60.54 ± 0.16 *c	68.05 ± 0.03 *d	65.89 ± 0.18 *e
Ash content	June	2.06 ± 0.20 a	1.71 ± 0.07 a	2.20 ± 0.22 b
	September	2.27 ± 0.01 d	1.15 ± 0.09 *c	1.37 ± 0.13 *c

* Asterisks denote statistically significant differences between June and September values (*p* < 0.05). Identical letters mean no statistically significant differences between the samples of different species (*p* > 0.05).

**Table 3:** The ANOVA results of the generalized linear mixed models (F-value and *p*-value) on the effect of species and month on chemical composition in the *Sphagnum* species: L—lipid fraction, WSF—water-soluble fraction, PS—pectin substances, HF—hemicellulose fraction, LLS—lignin-like substances, C—cellulose, AC—ash content.

Factor	df	LF		WSF		PS		HF		LLS		C		AC	
		F	*p*	F	*p*	F	*p*	F	*p*	F	*p*	F	*p*	F	*p*
Species	2	**57.2**	<0.001	**4.9**	0.028	2.1	0.169	**4.3**	0.040	2.9	0.091	1.4	0.276	**62.0**	<0.001
Season	1	**17.6**	0.001	**64.3**	<0.001	0.7	0.404	**74.0**	<0.001	**158.3**	<0.001	**1098.1**	<0.001	**65.2**	<0.001
Species × season	2	**8.6**	0.005	0.9	0.433	0.9	0.433	**17.4**	<0.001	2.8	0.101	**16.8**	<0.001	**33.7**	<0.001
R^2^			0.895		0.807		0.090		0.869		0.906		0.985		0.937

Statistically significant analysis results are in bold.

**Table 4:** Seasonal dynamics of the pigment complex and relative water content of *Sphagnum* species in the growing season 2023 (*n* = 3).

Sp.	Date	Chl *a* mg/g	Chl *b* mg/g	Chl (*a* + *b*) mg/g	Car mg/g	Chl *a*/*b*	Chl/Car	LHC, %	RWC, %
*S. lindbergii*	17.05	**0.41 ± 0.06**	**0.20 ± 0.02**	**0.60 ± 0.07**	**0.19 ± 0.02**	**2.08 ± 0.17**	3.10 ± 0.18	**72 ± 4.1**	1889
	14.06	**0.57 ± 0.03**	**0.28 ± 0.02**	**0.85 ± 0.05**	**0.26 ± 0.01**	**2.06 ± 0.06**	3.24 ± 0.14	**72 ± 1.3**	1969
	18.07	**0.47 ± 0.05**	**0.23 ± 0.02**	**0.70 ± 0.07**	**0.22 ± 0.02**	**2.04 ± 0.02**	3.17 ± 0.03	**72 ± 0.6**	1820
	17.08	0.70 ± 0.07 *	0.34 ± 0.02 *	1.05 ± 0.09 *	0.31 ± 0.01 *	**2.04 ± 0.13**	3.38 ± 0.23	**72 ± 3.0**	2280
	19.09	**0.49 ± 0.07**	**0.23 ± 0.02**	**0.72 ± 0.10**	**0.21 ± 0.02**	**2.16 ± 0.12**	3.36 ± 0.08	**70 ± 2.5**	2606
	18.10	0.40 ± 0.06 *	0.18 ± 0.02 *	0.58 ± 0.08 *	0.18 ± 0.02 *	**2.14 ± 0.15**	3.27 ± 0.07	**70 ± 3.4**	2585
*S. fuscum*	17.05	0.16 ± 0.04	0.08 ± 0.02	0.23 ± 0.06	0.11 ± 0.02	1.96 ± 0.04	2.21 ± 0.05	74 ± 1.0	974
	14.06	0.16 ± 0.04	0.08 ± 0.02	0.25 ± 0.06	0.16 ± 0.02	1.97 ± 0.04	1.57 ± 0.17 *	74 ± 1.0	1030
	18.07	0.13 ± 0.01	0.07 ± 0.01	0.20 ± 0.02	0.08 ± 0.004	1.96 ± 0.03	2.39 ± 0.14	74 ± 0.8	1045
	17.08	0.13 ± 0.04	0.07 ± 0.02	0.20 ± 0.06	0.09 ± 0.02	1.87 ± 0.09	2.08 ± 0.21	77 ± 2.3	855
	19.09	0.16 ± 0.05	0.08 ± 0.02	0.25 ± 0.07	0.10 ± 0.02	1.99 ± 0.08	2.41 ± 0.15	74 ± 1.9	1020
	18.10	0.20 ± 0.02	0.11 ± 0.01	0.31 ± 0.03	0.12 ± 0.02	1.91 ± 0.08	2.66 ± 0.29 *	76 ± 2.1	1108
*S. divinum*	17.05	0.50 ± 0.06	**0.24 ± 0.02**	0.74 ± 0.08	**0.24 ± 0.02**	**2.11 ± 0.07**	3.14 ± 0.05	**71 ± 1.7**	1244
	14.06	0.33 ± 0.03	**0.18 ± 0.02**	0.51 ± 0.06	**0.20 ± 0.03**	**1.81 ± 0.06**	2.56 ± 0.06 *	**78 ± 1.8**	1084
	18.07	0.39 ± 0.04	**0.20 ± 0.02**	0.59 ± 0.06	**0.19 ± 0.02**	**1.98 ± 0.06**	3.03 ± 0.05	**74 ± 1.5**	546
	17.08	0.64 ± 0.05 *	0.32 ± 0.02 *	0.96 ± 0.07 *	0.28 ± 0.03 *	**2.03 ± 0.04**	3.42 ± 0.11 *	**73 ± 0.9**	1243
	19.09	0.46 ± 0.01	**0.22 ± 0.01**	0.68 ± 0.02	**0.22 ± 0.01**	**2.10 ± 0.08**	3.06 ± 0.14	**71 ± 1.9**	1328
	18.10	0.29 ± 0.06 *	0.14 ± 0.02 *	0.43 ± 0.08 *	0.15 ± 0.03 *	**2.11 ± 0.10**	2.92 ± 0.08	**71 ± 2.4**	1315
*S. squarrosum*	20.05	**0.55 ± 0.07**	**0.23 ± 0.04**	**0.78 ± 0.11**	**0.24 ± 0.03**	2.43 ± 0.06	3.26 ± 0.10	64 ± 1.0	1815
	14.06	0.30 ± 0.05 *	0.13 ± 0.01 *	0.43 ± 0.06 *	0.14 ± 0.02 *	2.30 ± 0.11	3.20 ± 0.14	67 ± 2.2	2190
	18.07	**0.38 ± 0.06**	**0.18 ± 0.03**	**0.56 ± 0.09**	0.14 ± 0.02 *	2.08 ± 0.06 *	4.07 ± 0.07	72 ± 1.4 *	1181
	17.08	0.91 ± 0.12 *	0.42 ± 0.05 *	1.33 ± 0.17 *	0.41 ± 0.05 *	2.18 ± 0.06	3.26 ± 0.03	69 ± 1.3	733
	19.09	**0.50 ± 0.12**	**0.19 ± 0.04**	**0.69 ± 0.16**	**0.21 ± 0.04**	2.72 ± 0.03 *	3.29 ± 0.11	59 ± 0.5 *	922
	18.10	**0.65 ± 0.02**	**0.25 ± 0.01**	**0.90 ± 0.03**	**0.24 ± 0.01**	2.60 ± 0.05	3.74 ± 0.09	61 ± 0.9	1141

* Asterisks denote statistically significant differences between month values within a species (*p* < 0.05). Bold means no statistically significant differences between species when comparing samples formed by all values obtained during the study period, without dividing by months (*p* > 0.05).

**Table 5:** The ANOVA results of the generalized linear mixed models on the effect of species and month on pigment complex in the *Sphagnum* species.

Figure	*df*	Chl *a*		Chl *b*		Car		*a*/*b*		Chl/Car		LHC	
		F	*p*	F	*p*	F	*p*	F	*p*	F	*p*	F	*p*
Species	3	**163.0**	<0.001	**187.2**	<0.001	**103.0**	<0.001	**94.7**	<0.001	**304.7**	<0.001	**79.9**	<0.001
Month	5	**31.3**	<0.001	**47.3**	<0.001	**33.8**	<0.001	**16.1**	<0.001	**25.4**	<0.001	**13.1**	<0.001
Species × month	15	**12.2**	<0.001	**15.9**	<0.001	**14.7**	<0.001	**5.9**	<0.001	**11.9**	<0.001	**4.6**	<0.001
*R* ^2^			0.919		0.935		0.905		0.858		0.944		0.832

Statistically significant analysis results are in bold.

**Table 6:** The results of the Spearman’s correlation test on the environmental control over *Sphagnum* pigment complex. The table contains statistically significant correlation coefficients (*p* < 0.05).

Species	Meteorological Factor	Chl *a*	Chl *b*	Car	*a*/*b*	Chl/Car	LHC
*S. lindbergii*	Temperature	0.69	0.73	0.69			
	Precipitation						
	BWL	−0.69	−0.73	−0.69			
	Photoperiod						
	RWC					0.56	
*S. fuscum*	Temperature	−0.59	−0.53				
	Precipitation			0.61			
	BWL	0.59	0.53				
	Photoperiod					−0.66	
	RWC						
*S. divinum*	Temperature	0.57	0.60	0.51		0.55	
	Precipitation	−0.56	−0.56			−0.70	
	BWL	−0.66	−0.66	−0.60		−0.55	
	Photoperiod				−0.66		0.66
	RWC				0.65		−0.65
*S. squarrosum*	Temperature				−0.62		0.62
	Precipitation						
	BWL						
	Photoperiod	−0.69	−0.59	−0.61	−0.66		0.66
	RWC	−0.72	−0.66	−0.66			

**Table 7:** Weather and climatic data of the study period (May-October 2023).

Indicator		Sampling Day					
		17.05	14.06	18.07	17.08	19.09	18.10
Mean temperature for 10 days before sampling [43], °C		9.72	9.87	15.49	19.78	12.67	2.99
Sum of precipitation for 10 days before sampling, mm [43]		0.1	40.4	2.1	15.8	31.4	32.7
Water table level, cm	bog sites (1, 2)	−11	−12	−14	−16	−13	−8
	forest sites (3, 4)	−25	−36	−72	−80	−74	−15
Length of daylight, hh.mm [44]		18.47	21.21	19.39	16.17	12.39	9.29

**Table 8:** Characteristics of focal species. Data about hydration and trophic group for *Sphagnum* species is provided in accordance with Babeshina & Zverev (2010) [45].

Species	Section	Hydration Group	Trophic Group
*Sphagnum lindbergii* Schimp.	Cuspidata	hypohydrophyte	mesooligotrophophyte
*Sphagnum fuscum* (Schimp.) Klinggr	Acutifolia	hydromesophyte	orthooligotrophophyte
*Sphagnum divinum* Flatberg & K. Hassel	Sphagnum	hemihydrophyte	orthooligotrophophyte
*Sphagnum squarrosum* Crome	Squarrosa	hypohydrophyte	mesoeutrophophyte

## Data Availability

The raw data supporting the conclusions of this article will be made available by the authors on request.

## References

[1] Glime J.M. (2017). Chapter 2—Life Cycles and Morphology. Bryophyte Ecology. Volume 1: Physiological Ecology.

[2] Gorham E. (1991). Biogeochemistry: Its origins and development. Biogeochemistry.

[3] Wieder R.K., Vitt D.H., Benscoter B.W., Wieder R.K., Vitt D.H. (2006). Peatland and the boreal forest. Boreal Peatland Ecosystems.

[4] Heck M.A., Lüth V.M., van Gessel N., Krebs M., Kohl M., Prager A., Joosten H., Decker E.L., Reski R. (2021). Axenic In Vitro cultivation of 19 peat moss (*Sphagnum* L.) species as a resource for basic biology, biotechnology, and paludiculture. New Phytol..

[5] Asakawa Y., Ludwiczuk A. (2013). Bryophytes: Liverworts, mosses, and hornworts: Extraction and isolation procedures. Methods Mol. Biol..

[6] Goffinet B., Shaw A.J. (2008). Bryophyte Biology.

[7] Longton R.E. (1984). The role of bryophytes in terrestrial ecosystems. J. Hattori Bot. Lab..

[8] Michaelis D. (2012). Die *Sphagnum*-Arten der Welt. Taxon.

[9] Comis D. (1992). Miracle moss: Add water and watch it grow. Agric. Res..

[10] Proctor M.C.F. (2000). The bryophyte paradox: Tolerance of desiccation, evasion of drought. Plant Ecol..

[11] Titus J.E., Wagner D.J. (1984). Carbon balance for two *Sphagnum* mosses: Water balance resolves a physiological paradox. Ecology.

[12] Andrus R.E., Wagner D.J., Titus J.E. (1983). Vertical zonation of *Sphagnum* mosses along hummock-hollow gradients. Can. J. Bot..

[13] Popper Z.A., Fry S.C. (2003). Primary cell wall composition of bryophytes and charophytes. Ann. Bot..

[14] Glime J.M. (2017). Chapter 7—Water Relations. Bryophyte Ecology. Volume 1: Physiological Ecology.

[15] Gaudig G., Krebs M., Prager A., Wichmann S., Barney M., Caporn S.J.M., Emmel M., Fritz C., Graf M., Grobe A. (2017). Sphagnum farming from species selection to the production of growing media: A review. Mires Peat.

[16] Limpens J., Granath G., Gunnarsson U., Aerts R., Bayley S., Bragazza L., Bubier J., Buttler A., van den Berg L.J.L., Frances A.-J. (2011). Climatic Modifiers of the Response to Nitrogen Deposition in Peat-forming *Sphagnum* Mosses: A Meta-analysis. New Phytol..

[17] Loisel J., Gallego-Sala A.-V., Yu Z. (2012). Global-Scale Pattern of Peatland Sphagnum Growth Driven by Photosynthetically Active Radiation and Growing Season Length. Biogeosciences.

[18] Robroek B.J.M., Limpens J., Breeuwer A., Crushell P.H., Schouten M.G.C. (2007). Interspecific competition between *Sphagnum* mosses at different water tables. Funct. Ecol..

[19] Robroek B.J.M., Limpens J., Breeuwer A., Schouten M.G.C. (2007). Effects of water level and temperature on performance of four *Sphagnum* mosses. Plant Ecol..

[20] Boron D.J., Evans E.W., Peterson J.M. (1987). An overview of peat research, utilization, and environmental considerations. Int. J. Coal Geol..

[21] Lishtvan I.I., Bazin E.T., Gamayunov N.I., Terentyev A.A. (1989). Physics and Chemistry of Peat.

[22] Filippova N.V., Kosykh N.P., Filippov I.V., Niyazova A.V. (2023). Annual growth and primary production of sphagnum in raised bog Mukhrino (four-year observations: 2019–2022). Environ. Dyn. Glob. Clim. Chang..

[23] Mironov V.L., Kuznetsov O.L., Kantserova L.V., Kutenkov S.A., Ignashov P.A., Talbonen E.L., Vasyuta V.S., Svirida A.N. (2023). Comparison of linear increments and annual production of *Sphagnum* mosses obtained using three research methods (Koivulam-Bisuo mire system, southern Karelia). Trans. Karelian Res. Cent. Russ. Acad. Sci..

[24] Grabovik S.I., Kuznetsov O.L. (2016). Growth and productivity of cenopopulations of *Sphagnum* mosses in natural and transformed mires of Karelia. Trans. Karelian Res. Cent. Russ. Acad. Sci..

[25] Clymo R.W. (1970). The Growth of Sphagnum: Methods of Measurement. J. Ecol..

[26] Skillman J.B., Griffin K.L., Earll S., Kusama M., Pirajan J.C.M. (2011). Photosynthetic Productivity: Can Plants Do Better?. Thermodynamics—Systems in Equilibrium and Non-Equilibrium.

[27] Gunnarsson U. (2005). Global Patterns of *Sphagnum* Productivity. J. Bryol..

[28] Goncharova I.A. (2005). On the question of the structure of *Sphagnum* sod. Contemp. Probl. Ecol..

[29] Glime J.M. (2017). Chapter 9—Light. Bryophyte Ecology. Volume 1: Physiological Ecology.

[30] Limpens J., Bohlin E., Nilsson M.B. (2017). Phylogenetic or environmental control on the elemental and organo-chemical composition of Sphagnum mosses?. Plant Soil.

[31] Orlov D.S., Sadovnikova L.K. (2005). Soil organic matter and protective functions of humic substances in the biosphere, in Use of Humic substances to remediate Polluted Environments: From theory to practice. NATO Sci. Ser. IV Earth Environ. Sci..

[32] Bryan L., Shaw R., Schoonover E., Koehl A., DeVries-Zimmerman S., Philben M. (2024). Sphagnan in *Sphagnum*-dominated peatlands: Bioavailability and effects on organic matter stabilization. Biogeochemistry.

[33] Klavina L., Springe G. (2018). Seasonal changes of chemical composition in boreonemoral moss species. Environ. Exp. Biol..

[34] Pipes G.T., Yavitt J.B. (2022). Biochemical components of *Sphagnum* and persistence in peat soil. Can. J. Soil Sci..

[35] Volkova E.M., Akatova E.V., Boikova O.I., Khlytin N.V. (2019). The chemical and microbiological aspects of peat-forming processes on karst mires of Tula region. GeoScience.

[36] Aldous A.R. (2002). Nitrogen translocation in *Sphagnum* mosses: Effects of atmospheric nitrogen deposition. New Phytol..

[37] Glime J.M. (2017). Chapter 12—Productivity. Bryophyte Ecology. Volume 1: Physiological Ecology.

[38] Dymova O.V., Golovko T.K. (2018). Photosynthetic pigments: Functioning, ecology and biological activity. Bull. Ufa Sci. Cent. Wounds.

[39] Rice S.K. (1995). Patterns of Allocation and Growth in Aquatic *Sphagnum* Species. Can. J. Bot..

[40] Sestak Z. (1966). Limitation for finding a linear relashionship between chlorophyll content and photosynthetic activity. Biol. Plant..

[41] Skre O., Oechel W.C. (1981). Moss functioning in different taiga ecosystems in interior Alaska: I. Seasonal, phenotypic, and drought effects on photosynthesis and response patterns. Oecologia.

[42] Shakhov A.A., Korovin A.I. (1958). On the ecological characteristics of light assimilation. Fed. Res. Cent. Kola Sci. Cent. Russ. Acad. Sci..

[43] Federal Service for Hydrometeorology and Environmental Monitoring. http://meteo.ru/.

[44] World-Weather. https://world-weather.ru/.

[45] Babeshina L.G., Zverev A.A. (2010). Estimation of conditions of habitats of Sphagnum mosses in West-Siberian plain: Soil fertility factor. Tomsk. State Univ..

[46] Noskova M.G. (2016). Field Atlas-Identifier of Sphagnum Mosses of the Taiga Zone of European Russia.

[47] Churakova E.Y. (2002). Mosses of the taiga zone of the Arkhangelsk province (Northern European Russia). Arctoa.

[48] Hassel K., Kyrkjeeide M.O., Yousefi N., Presto T., Stenoien H.K., Shaw J.A. (2018). *Sphagnum divinum* (sp. nov.) and *S. medium* Limpr. and their relationship to *S. magellanicum* Brid. J. Bryol..

[49] Mironov V.L. (2017). On the potential of the method of geotropic curvatures for the study of growth in peat mosses. Trans. Karelian Res. Cent. Russ. Acad. Sci..

[50] Hajek T., Vicherova E. (2014). Desiccation Tolerance of *Sphagnum* Revisited: A Puzzle Resolved. Plant Biol..

[51] Laing C.G., Granath G., Belyea L.R., Alton K.E., Rydin H. (2014). Tradeoffs and Scaling of Functional Traits in *Sphagnum* as Drivers of Carbon Cycling in Peatlands. Oikos.

[52] Bengtsson F., Granath G., Cronberg N., Rydin H. (2020). Mechanisms behind Species-Specific Water Economy Responses to Water Level Drawdown in Peat Mosses. Ann. Bot..

[53] Zubov I.N., Orlov A.S., Popov A.N., Ponomareva T.I., Losyuk G.N. (2022). Evaluation of the Oil Absorption Capacity and Calorific Value of High-Moor Peat from the European North of Russia. Solid Fuel Chem..

[54] Babeshina L.G., Gorina Y.V., Kolokolova A.P., Krasnov E.A., Karpova M.R. (2010). Research of polysaccharides of some species *Sphagnum* L.. J. Sib. Fed. Univ. Chem..

[55] Lichtenthaler H.K. (1987). Chlorophylls and carotenoids—Pigments of photosynthetic biomembranes. Methods Enzymol..

[56] Dymova O.V., Golovko T.K. (2019). Photosynthetic Pigments in Native Plants of the Taiga Zone at the European Northeast Russia. Russ. J. Plant Physiol..

[57] Van De Koot W.Q.M., Msonda J., Olver O.P., Doonan J.H., Nibau H. (2024). Variation in Water-Holding Capacity in Sphagnum Species Depends on Both Plant and Colony Structure. Plants.

[58] Kuttim M., Kuttim L., Ilomets M., Laine A.M. (2020). Controls of *Sphagnum* Growth and the Role of Winter. Ecol. Res..

[59] Ivanov L.A., Ronzhina D.A., Yudina P.K., Kalashnikova I.V., Ivanova L.A., Zolotareva N.V. (2020). Seasonal dynamics of the chlorophyll and carotenoid content in the leaves of steppe and forest plants on species and community level. Russ. J. Plant Physiol..

[60] Nasledov A.D. (2008). SPSS 15: Professional Statistical Data Analysis.

